# Comparison between 100-g glucose tolerance test and two other screening tests for gestational diabetes: combined fasting glucose with risk factors and 50-g glucose tolerance test

**DOI:** 10.1590/S1516-31802006000100002

**Published:** 2006-01-05

**Authors:** Wilson Ayach, Roberto Antonio Araújo Costa, Iracema de Mattos Paranhos Calderon, Marilza Vieira Cunha Rudge

**Keywords:** Blood glucose, Gestational diabetes, Glucose tolerance test, Hyperglycemia, Diabetes mellitus, Glicemia, Diabetes gestacional, Teste de tolerância a glucose, Hiperglicemia, Diabetes melito

## Abstract

**CONTEXT AND OBJECTIVE:**

Lack of consensus about which screening tests to use for gestational diabetes mellitus (GDM) and difficulties in performing the gold-standard diagnostic test, the 100-g glucose tolerance test (100-g GTT), justify comparison with alternatives. The aim was to compare this with two other screening tests: combined fasting glucose with risk factors (FG + RF) and 50-g GTT.

**DESIGN AND SETTING:**

Prospective longitudinal cohort study in the Hospital School of Universidade Federal de Mato Grosso do Sul.

**METHODS:**

The three tests were performed independently on 341 pregnant women. Sensitivity (S), specificity (Sp), positive (PPV) and negative (NPV) predictive values, positive (PLR) and negative (NLR) likelihood ratios, and false-positive (FP) and false-negative (FR) rates obtained with FG + RF and 50-g GTT were compared with values from 100-g GTT. The average one-hour post-intake glucose levels (1hPG) with 50-g and 100-g were compared. Student's t test was used in the statistical analysis.

**RESULTS:**

FG + RF led more pregnant women (53.9%) to diagnostic confirmation than did 50-g GTT (14.4%). The tests were equivalent for S (86.4 and 76.9%), PPV (98.7 and 98.9%), NLR (0.3 and 0.27) and FR (15.4 and 23.1%). Average 1hPG values were similar: 50-g GTT = 106.8 mg/dl and 100-g GTT = 107.5 mg/dl.

**CONCLUSION:**

Diagnostic efficiency with simplicity, practicality and low cost make FG + RF more appropriate for screening for GDM. The equivalence of 1hPG allows a new, cheaper and less uncomfortable protocol to be proposed for screening and diagnosing GDM.

## RESUMO

**CONTEXTO E OBJETIVO:**

A falta de consenso sobre os protocolos de rastreamento e diagnóstico do diabetes gestacional, associada às dificuldades na realização do teste oral simplificado do diabete gestacional (o teste de tolerância a 100 g de glicose, considerado padrão-ouro) justificam a comparação com alternativas. O objetivo deste trabalho é comparar o teste padrão-ouro a dois testes de rastreamento: associação de glicemia de jejum e fatores de risco (GJ + FR) e o teste oral simplificado de tolerância a 50 g de glicose (TTG 50 g), com o teste de tolerância a 100 g de glicose (TTG 100 g).

**TIPO DE ESTUDO E LOCAL:**

Estudo de coorte longitudinal, prospectivo, realizado no Serviço de Ginecologia e Obstetrícia do Hospital Universitário da Universidade Federal de Mato Grosso do Sul.

**MÉTODOS:**

341 gestantes foram submetidas aos três testes. Calcularam-se os índices de sensibilidade (S), especificidade (E), valores preditivos (VPP e VPN), razões de probabilidade (RPP e RPN) e resultados falsos (FP e FN), positivos e negativos da associação GJ + FR e do TTG 50 g em relação ao TTG 100 g. Compararam-se as médias das glicemias de uma hora pós-sobrecarga (1hPS) com 50 e 100 g. Na análise estatística, empregou-se o teste *t* de Student, com limite de significância de 5%.

**RESULTADOS:**

A associação GJ + FR encaminhou mais gestantes (53,9%) para a confirmação diagnóstica que o TTG 50 g (14,4%). Os dois testes foram equivalentes nos índices de S (86,4 e 76,9%), VPN (98,7 e 98,9%), RPN (0,3 e 0,27) e FN (15,4 e 23,1%). As médias das glicemias 1hPS foram semelhantes, 106,8 mg/dl para o TTG 50 g e 107,5 mg/dl para o TTG 100 g.

**CONCLUSÕES:**

Os resultados da eficiência diagnóstica associados à simplicidade, praticabilidade e custo referendaram a associação GJ + FR como o mais adequado para o rastreamento. A equivalência das glicemias de 1hPS permitiram a proposição de um novo protocolo de rastreamento e diagnóstico do diabete gestacional, com menores custo e desconforto.

## INTRODUCTION

Gestational diabetes mellitus (GDM) is defined as any degree of glucose intolerance with onset or first recognition during pregnancy. ^1^ This definition and the higher risk of maternal and fetal complications brought about by maternal hyperglycemia make it essential to diagnose diabetes mellitus during pregnancy.^2-4^ Early diagnosis of this complication and appropriate treatment aimed at tight control over maternal glucose levels may positively influence the perinatal outcome.^2-4^

Gestational diabetes mellitus is a group of diseases that result from deficiencies in insulin action and/or insulin secretion. Even if first detected during pregnancy, the possibility of its preexistence cannot be disregarded. Furthermore, the intrinsic characteristics of glucose tolerance tests hinder the reaching of consensus regarding the most effective method for screening and diagnosing this disease.^5^

With the aim of diagnosing gestational diabetes, two steps have been standardized: screening and diagnostic confirmation.^4^ The establishment of a diagnosis is, per se, an imperfect process. The use of a diagnostic test, whether it is a clinical or a laboratory test, enhances the probability but brings no certainty that a diagnosis is accurate. The diagnostic test that provides a faithful indication of the truth, which is also known as the gold standard, is not always perfect or available.^6-8^

The 100-g glucose tolerance test (100-g GTT) is the most widely used method for diagnosing diabetes during pregnancy. It must be performed at 24-28 weeks of gestation, and it measures maternal glucose levels while fasting, as well as one, two and three hours after glucose intake. The diagnosis of gestational diabetes is confirmed when two or more measurements equal or exceed the plasma glucose values established by O'Sullivan & Mahan^9^: 95, 180, 155 and 140 mg/dl, under fasting conditions and one, two and three hours after intake, respectively.^1^ Despite being widely known and used, 100-g GTT is not perfect. The number of measurements needed for determining diagnostic confirma- tion,^10,11^ the amount of oral glucose intake, and the need to consider the perinatal prognosis in order to determine threshold values are still under debate.^12,13^

Alternatively, the diagnosis can be made using a 75-g glucose load and the glucose threshold values listed for fasting, 1 h, and 2 h (Table 2); however, this test is not as well validated for detection of at-risk infants or mothers as the 100-g OGTT. The World Health Organization (WHO) recommends a one-step approach to the screening and diagnosis of gestational diabetes: a 75-g glucose tolerance test (75-g GTT) on all pregnant women between the 24^th^ and 28^th^ weeks of gestation. The diagnosis is confirmed when fasting glucose equals or exceeds 126 mg/dl or when the two-hour post-intake glucose equals or exceeds 200 mg/dl. Fasting glucose > 105 and < 126 mg/dl and/or two-hour post-intake glucose > 140 and < 200 mg/dl characterize glucose intolerance.^11^

Abnormal 75-g GTT results show strong correlation with macrosomia,^14,15^ high cesarean section rates,^14,16^ and poor perinatal outcomes (the higher the two-hour post-intake glucose levels, the poorer the outcome).^16^ These findings explain the modifications to the American Diabetes Association (ADA) recommendations that have been made,^1^ which now support 75-g GTT (with glucose threshold values of 95, 180, 155 and 140 mg/dl, under fasting conditions and 1, 2 and 3 hours after load, respectively), as an alternative to 100-g GTT for the diagnosing of gestational diabetes. The ADA also considers the values provided by these two methods (100 and 75-g GTT) to be similar.

Since the performing of these diagnostic tests in all pregnancies is very expensive, effective screening for gestational diabetes is vital for increasing the cost-benefit ratio. The ADA recommends that women at high risk of GDM (age ≥ 25, body mass index, BMI, before pregnancy ≥ 27 kg/m^2^, family or personal history of diabetes, and membership of an ethnic group with high prevalence of GDM) should undergo 50-g GTT during the second trimester of gestation, with a plasma glucose threshold value of 140 mg/dl one hour after glucose load intake under nonfasting conditions.^1^

Another screening test, proposed by Rudge & De Luca^17^ considers the combination of fasting glucose (FG) with the presence of risk factors (FG + RF). This screening is positive if fasting glucose ≥ 90 mg/dl and/or risk is confirmed (age ≥ 30 years, BMI before pregnancy ≥ 27 kg/m^2^, family history of diabetes, gestational diabetes, macrosomia, unexplained fetal loss and fetal malformation). According to these authors, this screening method provides results similar to those of the 50-g GTT, is easier to perform, costs less and is more comfortable for patients, yet leads a larger number of women to diagnostic confirmation.^17^ A prospective study, in which these two screening approaches were used in the same population of pregnant women, showed that, using the FG + RF combination, the sensitivity was 83.7% and the negative predictive value (NPV) was 95.8%.^18^

Most of the guidelines that endorse 50-g GTT as the gold standard test for the screening of gestational diabetes highlight its ability to predict the future onset of diabetes mel- litus^19^ as well as the recurrence of gestational diabetes,^20^ and also its strong correlation with the most widely used 100-g GTT.^21^ On the other hand, the ease of performance and low cost of the FG + RF combination, characteristics that are essential for a screening test^5^ and appropriate for Brazilian realities, have caused this method to be recommended in the Guidelines for Prenatal Care issued by the Brazilian Ministry of Health.^22^

Comparison of FG + RF and 50-g GTT with 100-g GTT may enable determination of the validity of each of these tests. The present study had the aim of comparing two screening methods with the most used test for the diagnosing of gestational diabetes. Thus, the sensitivity, specificity, positive and negative predictive values and likelihood ratios were calculated for the tests. Furthermore, the average glucose values one hour after intake of 50 g and 100 g of glucose and the cost of each laboratory test used for screening and diagnosing gestational diabetes were assessed.

## SUBJECTS AND METHODS

This was a prospective longitudinal cohort study that followed the validation design for gestational diabetes mellitus screening tests. The minimum sample size was calculated considering the population to be finite, the previous incidence of gestational diabetes to be a nominal variable, and the sample error to be 2.5%. The estimated sample size was 282, and 341 pregnant women were in fact analyzed.^23^ The study was conducted in the Hospital School of Universidade Federal de Mato Grosso do Sul (HU-UFMS) between July, 1997 and December, 1999.

All pregnant women with no previous history of diabetes mellitus who sought prenatal care in HU-UFMS during the first half of their pregnancies were considered eligible. After giving their informed consent, the patients were assessed according to the study protocol. This included the obtaining of the patients’ medical history, calculation of gestational age, obstetric ultrasound scan, measurement of fasting glucose levels, and 50-g GTT and 100-g GTT performed between the 24^th^ and 28^th^ weeks of pregnancy. The patients’ histories were taken on their first visit to prenatal care in order to identify risk factors: age ≥ 30 years, pre-gestational BMI ≥ 27 kg/m^2^, personal history of gestational diabetes, family history of diabetes mellitus, macrosomia, fetal death with no apparent cause, recurrent miscarriages and malformation.^17^

Plasma glucose levels were measured using the glucose-oxidase enzyme method, with a coefficient of variation < 5%.^24^ Fasting glucose was evaluated after 8 to 12 hours of fasting on two occasions: before the 20^th^ week of pregnancy and when the 50-g GTT was performed between the 24^th^ and 28^th^ weeks of gestation.^1^

FG + RF screening was considered positive when fasting plasma glucose levels equaled or exceeded 90 mg/dl and/or any risk factor for gestational diabetes was pres- ent.^17^ The 50-g GTT screening was considered positive when the one-hour post-intake glucose level was ≥ 140 mg/dl.^1^ Regardless of the results from the screening tests, all the pregnant women also underwent 100-g GTT, which was considered positive when two or more values equaled or exceeded 95, 180, 155 and 140 mg/dl, under fasting conditions, and one, two and three hours after glucose intake, respectively.^4^

Participation in the study was terminated according to the following criteria: failure to perform or finish the screening and diagnostic tests, withdrawal of consent or premature termination of the pregnancy.

The screening tests were compared with regard to their costs and one-hour post-intake glucose levels. Costs were determined in reais (R$) (two Reais ≅ two American dollars), considering the real cost for the laboratory and the amount paid by the Brazilian National Health System (Sistema Único de Saúde, SUS).

In addition, the efficiency of each test was compared with that of the 100-g GTT. The diagnostic efficiency was evaluated by means of the sensitivity (S), specificity (Sp), positive (PPV) and negative (NPV) predictive values, and positive (PLR) and negative (NLR) likelihood ratios.^6-8^

Comparison of the diagnostic efficiency between the tests was done using Student's t test. The average post-intake glucose levels obtained one hour after receiving 50 g and 100 g (1hPG) and the differences between these and the fasting glucose levels (1hPG–FG) were evaluated using the paired Student's t test. The statistical significance was set at 5% (p < 0.05).^6-8^

This study was approved by the Research Ethics Committees of Universidade Federal de Mato Grosso do Sul (UFMS) and Faculdade de Medicina de Botucatu, Universidade Estadual Paulista (FMB/Unesp).

## RESULTS

Among the 465 pregnant women invited, 460 consented to participate in this study. However, 119 of them were unable to participate as they did not meet the inclusion criteria, due to miscarriage (n = 7), pseudocyesis (n = 1), premature childbirth (n = 2), fetal death (n = 2), intolerance to oral glucose (n = 3), and dropout before performing the 50-g GTT (n = 86) or 100-g GTT (n = 18). By the end of the study period, 341 women had been evaluated.

Demographic analysis showed that 15.8% of the pregnant women were ≥ 30 years old. Pre-gestational BMI ≥ 27 kg/m^2^ was observed in 14.4% of the cases. More than half of the subjects were white (61.0%) and 43.3% were primigravida (Table 1).

**Table 1:** Number (n) and percentage (%) of pregnant women according to age, body mass index (BMI), race and number of pregnancies

	n	%
Age ≥ 30 years	54	15.8
BMI ≥ 27 kg/m^2^	49	14.4
White	208	61.0
Primigravida	148	43.4

FG + RF screening was positive for 184 women (53.9%) and the 50-g GTT was abnormal in 54 cases (15.8%). The 100-g GTT was normal in 328 subjects (96.2%) and confirmed the diagnosis of gestational diabetes mellitus in 13 cases (3.8%) (Table 2).

**Table 2:** Number (n) and percentage (%) of pregnant women according to the results of the screening tests, FG + RF (fasting glucose plus risk factors) and 50-g GTT (glucose tolerance test), in comparison with 100-g GTT

	100-g GTT			
	Abnormal		Normal	
	n	%	n	%
FG + RF positive	11	84.6	173	52.7
FG + RF negative	02	15.4	155	47.3
50-g GTT positive	10	76.9	44	13.4
50-g GTT negative	03	23.1	284	86.6
**Total**	**13**	**3.8**	**328**	**96.2**

FG + RF screening was positive for gestational diabetes in 84.6% and the 50-g GTT was abnormal in 76.9% of the cases with a confirmed diagnosis for gestational diabetes. When FG + RF was negative, half of the diagnostic tests (47.3%) were normal and 50-g GTT was negative in 86.6% of the women with a normal 100-g GTT (Table 2).

FG + RF screening was positive for gestational diabetes in 84.6% and the 50-g GTT was abnormal in 76.9% of the cases with a confirmed diagnosis for gestational diabetes. When FG + RF was negative, half of the diagnostic tests (47.3%) were normal and 50-g GTT was negative in 86.6% of the women with a normal 100-g GTT (Table 2).

By comparing the other screening tests (FR + RF and 50-g GTT) with the 100-g GTT, it was confirmed that they showed similar and high sensitivities (86.4% and 76.9%, respectively) and negative predictive values (98.7% and 98.9%). The negative likelihood ratios (0.3 and 0.27) and the rates of false-negative results (15.4% and 23.1%) observed were low and also equivalent. The screening tests differed statistically in relation to specificity (Sp), positive predictive value (PPV), positive likelihood ratio (PLR) and false-positive results (FP). FG + RF showed lower Sp (47.3%), PPV (6.0%) and PLR (1.6%) than did 50-g GTT (86.6%, 18.5% and 5.7%, respectively). However, the rate of FP results was higher for FG + RF (52.7%) than for 50-g GTT (13.4%) (Table 3).

**Table 3:** Rate (%) and confidence interval (95% confidence interval, CI) of sensitivity (S), specificity (Sp), positive (PPV) and negative (NPV) predictive values, positive (PLR) and negative (NLR) likelihood ratio, false positive (FP) and false negative (FN) results for the FG + RF (fasting glucose plus risk factors) and 50-g GTT (glucose tolerance test), in comparison with 100-g GTT

	FR+RF		50-g GTT		p*
	%	95% CI	%	95% CI	
S	84.6	54.5 – 98.1	76.9	46.2 – 95.0	0.62
Sp	47.3	41.8 – 52.8	86.6	82.4 – 90.0	< 0.000001
PPV	6.0	3.0 – 10.0	18.5	9.3 – 31.4	0.0002
PNV	98.7	95.5 – 99.8	98.9	97.0 – 99.8	0.8
PLR	1.6	0.9 – 2.1	5.7	2.6 – 9.5	0.0002
NLR	0.3	0.03 – 1.1	0.27	0.05 – 2.6	0.9
FP	52.7	41.8 – 58.2	13.4	10.0 – 17.6	0.0002
FN	15.4	1.9 – 45.5	23.1	5.0 – 53.8	0.8

* *Student's t test.*

The average one-hour post-intake glucose levels were 106.8 mg/dl and 107.5 mg/dl after intakes of 50 g and 100 g, respectively. The differences between average one-hour post-intake and fasting glucose levels were 36.3 mg/dl for 50-g GTT and 30.8 mg/dl for 100-g GTT. These average values were statistically similar (Table 4).

**Table 4:** Mean (M) and standard deviation (SD) for fasting glucose (FG), one hour post-intake glucose level (1hPG) using 50-g GTT (glucose tolerance test) and 100-g GTT, and the difference between 1hPG and FG

	50-g GTT		100-g GTT		
	M	SD	M	SD	p*
FG	70.5	15.0	76.6	11.0	3.60
1hPG	106.8	29.6	107.5	30.8	0.27
1hPG–FG	36.3	34.5	30.8	34.0	0.10

* *Paired Student's t test.*

For the University Hospital Clinical Laboratory, the real costs of fasting glucose tests and 50-g GTT were R$ 1.85 and R$ 4.70, respectively. The amounts paid by SUS for these procedures were R$ 1.85 and R$ 3.63, respectively. Both fasting and 100-g GTT cost the laboratory R$ 5.70 and SUS paid R$ 10.00 for them. The real cost of a complete 100-g GTT was R$ 9.40 and SUS paid R$ 10.00 for it (Table 5).

**Table 5:** Real cost, amount paid by the Brazilian National Health System (*Sistema Único de Saúde*, SUS) and profit or loss to the laboratory, calculated in Reais (R$), for each laboratory test used in screening for and diagnosing gestational diabetes

	Real cost*	SUS	Profit/loss to laboratory
Fasting glucose level	1.85	1.85	0.00
50-g GTT	4.70	3.63	- 1.07
100-g GTT (two measurements)	5.70	10.00	+ 4.30
100-g GTT (complete)	9.40	10.00	+ 0.60

*GTT = glucose tolerance test.*

* *taking University Hospital Clinical Laboratory of Universidade Estadual de São Paulo (Unesp), as a reference.*

## DISCUSSION

The results obtained in this study showed that a greater number of pregnant women were led to diagnostic confirmation by the FG + RF screening method than by the 50-g GTT. By comparing FG + RF and the 50-g GTT with the gold standard test for the diagnosis of diabetes in pregnancy (100-g GTT), it was demonstrated that sensitivity, NPV, NLR and FN rates were equivalent. The FG + RF method provided higher rates of FN results, while the specificity rates, PPV and PLR were significantly higher for the 50-g GTT. Furthermore, the 50-g GTT and the one-hour post-intake 100-g GTT were confirmed to be statistically equivalent.

In the light of these results, which is the best method for screening gestational diabetes? The answer to this question lies in defining efficiency indexes, the characteristics inherent to the screening tests, and the realities of each healthcare service.

Sensitivity measures the proportion of individuals with a disease that are correctly identified by the test, while specificity measures the proportion of individuals without the disease who are correctly labeled disease-free by the test. These characteristics are inherent to a test and are not dependent on the prevalence of the disease. However, they may be influenced by the quality of the gold standard. Predictive value means the probability of the disease being either present when the test is positive (positive predictive value, PPV) or absent when the test is negative (negative predictive value, NPV) and depends on the test sensitivity and specificity as well as the prevalence of the disease. The likelihood ratio expresses the relationship between sensitivity and specificity. The positive likelihood ratio (PLR) shows the best test to use for ruling a disease in while the negative likelihood ratio (NLR) indicates which test should be used to rule it out. The rates of false-positives (FP) and false-negatives (FN) report the percentage of individuals who were incorrectly categorized by the screening test. Like predictive values, FP and FN rates are influenced by the test's sensitivity and specificity as well as the prevalence of the disease.^6-8^

In general, the use of highly sensitive tests is recommended at the beginning of a clinical investigation, in order to reduce the range of diagnostic possibilities, to rule out the disease tested, and whenever a failure in diagnosing may cause important complications. Tests of high specificity are used to confirm a diagnosis or whenever false-positive results may cause physical, emotional or financial harm.

The likelihood ratio alone can determine the best test to use if the consequences from a false-positive test are equal to the consequences from a false-negative test.^6-8^ When screening for gestational diabetes, false-negative results are more important than false-positives. In this situation, the best test to use is the one able to rule out the disease, i.e. with the highest rates of sensitivity, NPV and NLR. In the present study, the tests assessed showed statistically similar rates of sensitivity, NPV and NLR.

Besides these efficiency-related characteristics, the ideal screening test should be simple, practical, reproducible and inexpensive. These attributes can be recognized in the FG + RF combination: history-taking is inherent to clinical practice and the measurement of fasting glucose is routine in prenatal care.^22^ The major drawbacks presented by this method are its high rate of false-positives and low specificity, which lead a greater number of pregnant women to diagnostic confirmation. However, these disadvantages are outweighed by the low rate of false-negative results that was observed (suitable for a good screening test), and the consequences of undiagnosed gestational diabetes.

The 50-g GTT is considered to be the standard test for the screening of gestational diabetes. Its efficiency, already known and previously determined, was confirmed in this study.^1,18^ In addition to the characteristics inherent to a screening test, which were found to be similar to those of the FG + RF combination, the 50-g GTT has high specificity in comparison with the 100-g GTT. The similarity between the 50-g and the 100-g glucose tolerance tests was corroborated when their average one-hour post-intake results were compared. This equivalence with the 100-g GTT shows that the use of the 50-g GTT is much more appropriate for diagnosis than for screening. However, the intake of 50 g of glucose is not free of side effects, and its repetition during pregnancy is an unnecessary risk. Repetition of glucose tolerance tests has been associated with higher fetal weight and macrosomia in pregnant rats.^25^

In the present study, the results obtained confirmed that the attributes that are desirable for a good screening test are present in both the tests evaluated: high S and NPV, and low NLR and FN. Nonetheless, when these parameters are analyzed separately, they are insufficient for determining which test is better for screening for gestational diabetes in developing countries. Other characteristics must also be considered.

The ADA recommends the use of 50-g and 100-g glucose tolerance tests for screening for and diagnosing gestational diabetes. It costs the laboratory R$ 210.81 to perform these tests in populations at high risk of developing GDM, and this generates a deficit of R$ 28.06 per diagnosis made in relation to the SUS payment. The use of the FG + RF method costs R$ 181.57 per case of confirmed diabetes and is totally paid for by SUS. Therefore, the use of the FG + RF method and 100-g GTT to diagnose diabetes brings the benefit, among others, of being less costly.

**Figure 1:**
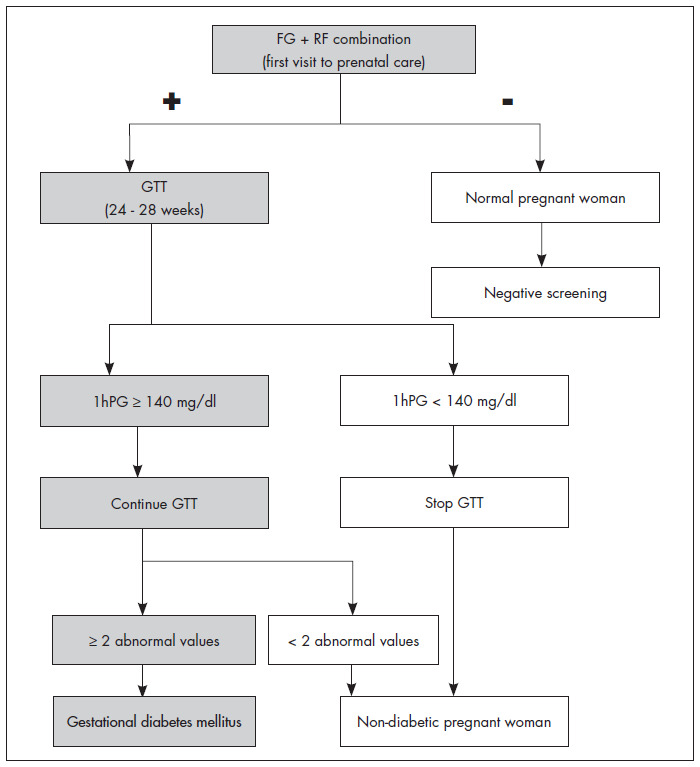
Proposal for a protocol for screening for and diagnosing gestational diabetes mellitus.

Considering the total cost (from screening to diagnostic confirmation), the equivalence between the 50-g GTT and 100-GTT, and the risk involved in repeating the tests, we propose a new protocol (Figure 1). This new protocol includes the use of a more practical and simpler screening test, the FG + RF combination. A negative result would classify the patient as low-risk and would be enough to rule out the disease, as demonstrated by the high NPV and NLR of the combination, in comparison with those of the 100-g GTT. Pregnant women at risk of developing gestational diabetes, as identified by positive FG + RF, would have to undergo diagnostic confirmation via the 100-g GTT between the 24^th^ and 28^th^ weeks of gestation. The greater number of pregnant women requiring diagnostic confirmation that would result from the low PPV and high FP rates provided by this screening test could be reduced by using the 100-g GTT itself.

*FG = fasting glucose; RF = risk factor; GTT = glucose tolerance test; 1hPG = one-hour post-intake glucose.*

While performing the 100-g GTT, the evaluation of the one-hour post-intake glucose would correspond to a new screening test: positive, when the glucose ≥ 140 mg/dl, or negative, when the glucose < 140 mg/dl. A positive result would identify that the pregnant women was at a high risk of developing diabetes and make it necessary to complete the 100-g GTT. A negative result would be enough to allow abnormal results from a complete 100-g GTT to be disregarded, thereby making it unnecessary to measure the glucose two or three hours after intake. By using this protocol, the real cost of each case of confirmed gestational diabetes would be R$ 138.88, and a profit of R$ 57.16 would be generated for the laboratory, considering the values paid by SUS.

According to this new protocol (Figure 1), two screening tests would be applied in series. The first would be simple, practical, accurate and cheap, and the second, despite the inconvenience of glucose intake, would form part of the diagnostic test. The benefits would include the performing of only one glucose tolerance test, the reduction of costs per diagnosed case and the assurance of less discomfort for pregnant women.

## CONCLUSION

The comparison of two screening tests for gestational diabetes, FG + RF and 50-g GTT, with the gold-standard diagnostic 100-g GTT allowed the following conclusions to be reached:

1. FG + RF screening leads a greater number of pregnant women to diagnostic confirmation than does the 50-g GTT.
2. The S, NPV, NLR and FN show that the two screening methods are similar.
3. These values, associated with the simplicity, practicality and cost of FG + RF, indicate that this is a more appropriate screening method for gestational diabetes.
4. The statistical equivalence between the one-hour post-intake levels using the 50-g and 100-g GTT makes it possible to propose a new, simpler, more practical and cheaper protocol for screening for and diagnosing gestational diabetes.
